# A 7-Year Brazilian National Perspective on Plasmid-Mediated Carbapenem Resistance in Enterobacterales, *Pseudomonas aeruginosa*, and *Acinetobacter baumannii* Complex and the Impact of the Coronavirus Disease 2019 Pandemic on Their Occurrence

**DOI:** 10.1093/cid/ciad260

**Published:** 2023-07-05

**Authors:** Carlos R V Kiffer, Thais F T Rezende, Daniela Testoni Costa-Nobre, Ana Sílvia Scavacini Marinonio, Lucas Hidemitsu Shiguenaga, Debora Nicole Oliveira Kulek, Lavinia Nery Villa Stangler Arend, Ivson Cassiano de Oliveira Santos, Bruna Ribeiro Sued-Karam, Claudio Marcos Rocha-de-Souza, Leticia Kraft, Andre Abreu, Renata Tigulini de Souza Peral, Ana Paula D’Alincourt Carvalho-Assef, Marcelo Pillonetto

**Affiliations:** Escola Paulista de Medicina (EPM), Universidade Federal de São Paulo—UNIFESP, São Paulo, Brazil; Infectious Diseases Discipline, Laboratório Especial de Microbiologia Clínica—LEMC/ALERTA, EPM, UNIFESP, São Paulo, Brazil; Escola Paulista de Medicina (EPM), Universidade Federal de São Paulo—UNIFESP, São Paulo, Brazil; Infectious Diseases Discipline, Laboratório Especial de Microbiologia Clínica—LEMC/ALERTA, EPM, UNIFESP, São Paulo, Brazil; Escola Paulista de Medicina (EPM), Universidade Federal de São Paulo—UNIFESP, São Paulo, Brazil; Escola Paulista de Medicina (EPM), Universidade Federal de São Paulo—UNIFESP, São Paulo, Brazil; Escola Paulista de Medicina (EPM), Universidade Federal de São Paulo—UNIFESP, São Paulo, Brazil; Laboratorio Central do Paraná - LACENPR, Secretaria de Estado da Saúde, Curitiba, Parana, Brazil; Escola de Medicina e Ciencias da Vida - EMCV, Pontifícia Universidade Católica do Paraná—PUCPR, Curitiba, Parana, Brazil; Laboratorio Central do Paraná - LACENPR, Secretaria de Estado da Saúde, Curitiba, Parana, Brazil; Escola de Medicina e Ciencias da Vida - EMCV, Pontifícia Universidade Católica do Paraná—PUCPR, Curitiba, Parana, Brazil; Instituto Oswaldo Cruz (IOC)—Fundação Instituto Oswaldo Cruz (FIOCRUZ), Rio de Janeiro, Brazil; Laboratório de Pesquisa em Infecção Hospitalar—LAPIH, Instituto Oswaldo Cruz, Rio de Janeiro, Brazil; Instituto Oswaldo Cruz (IOC)—Fundação Instituto Oswaldo Cruz (FIOCRUZ), Rio de Janeiro, Brazil; Laboratório de Pesquisa em Infecção Hospitalar—LAPIH, Instituto Oswaldo Cruz, Rio de Janeiro, Brazil; Instituto Oswaldo Cruz (IOC)—Fundação Instituto Oswaldo Cruz (FIOCRUZ), Rio de Janeiro, Brazil; Laboratório de Pesquisa em Infecção Hospitalar—LAPIH, Instituto Oswaldo Cruz, Rio de Janeiro, Brazil; Coordenação Geral de Laboratórios de Saúde Publica, Departamento de Articulação Estratégica de Vigilância em Saúde e Ambiente, Secretaria de Vigilância em Saúde e Ambiente, Ministério da Saúde, Brasilia, Distrito Federal, Brazil; Núcleo de Epidemiologia e Vigilância em Saúde, Instituto Oswaldo Cruz—FIOCRUZ, Brasilia, Distrito Federal, Brazil; Coordenação Geral de Laboratórios de Saúde Publica, Departamento de Articulação Estratégica de Vigilância em Saúde e Ambiente, Secretaria de Vigilância em Saúde e Ambiente, Ministério da Saúde, Brasilia, Distrito Federal, Brazil; Instituto Oswaldo Cruz (IOC)—Fundação Instituto Oswaldo Cruz (FIOCRUZ), Rio de Janeiro, Brazil; Laboratório de Pesquisa em Infecção Hospitalar—LAPIH, Instituto Oswaldo Cruz, Rio de Janeiro, Brazil; Laboratorio Central do Paraná - LACENPR, Secretaria de Estado da Saúde, Curitiba, Parana, Brazil; Escola de Medicina e Ciencias da Vida - EMCV, Pontifícia Universidade Católica do Paraná—PUCPR, Curitiba, Parana, Brazil

**Keywords:** carbapenemases, COVID-19, *bla*_NDM_, *bla*_KPC_, *bla*_OXA-23_

## Abstract

**Background:**

Carbapenemase production is a global public health threat. Antimicrobial resistance (AMR) data analysis is critical to public health policy. Here we analyzed carbapenemase detection trends using the AMR Brazilian Surveillance Network.

**Methods:**

Carbapenemase detection data from Brazilian hospitals included in the public laboratory information system dataset were evaluated. The detection rate (DR) was defined as carbapenemase detected by gene tested per isolate per year. The temporal trends were estimated using the Prais–Winsten regression model. The impact of COVID-19 on carbapenemase genes in Brazil was determined for the period 2015–2022. Detection pre- (October 2017 to March 2020) and post-pandemic onset (April 2020 to September 2022) was compared using the *χ*^2^ test. Analyses were performed with Stata 17.0 (StataCorp, College Station, TX).

**Results:**

83 282 *bla*_KPC_ and 86 038 *bla*_NDM_ were tested for all microorganisms. Enterobacterales DR for *bla*_KPC_ and *bla*_NDM_ was 68.6% (41 301/60 205) and 14.4% (8377/58 172), respectively. *P. aeruginosa* DR for *bla*_NDM_ was 2.5% (313/12 528). An annual percent increase for *bla*_NDM_ of 41.1% was observed, and a decrease for *bla*_KPC_ of −4.0% in Enterobacterales, and an annual increase for *bla*_NDM_ of 71.6% and for *bla*_KPC_ of 22.2% in *P. aeruginosa*. From 2020 to 2022, overall increases of 65.2% for Enterobacterales, 77.7% for ABC, and 61.3% for *P. aeruginosa* were observed in the total isolates.

**Conclusions:**

This study shows the strengths of the AMR Brazilian Surveillance Network with robust data related to carbapenemases in Brazil and the impact of COVID-19 with a change in carbapenemase profiles with *bla*_NDM_ rising over the years.

## Teaser

Seven-year surveillance in Brazil showed a >60% increase in resistance genes during the COVID-19 pandemic, especially *bla*_NDM_ in Enterobacterales and *Pseudomonas aeruginosa*. *bla*_KPC_ in Enterobacterales and *bla*_SPM_ in *P. aeruginosa* decreased. These data contribute to Antimicrobial Resistance public policies.

Antimicrobial resistance (AMR) has become a threat to public health due to the growing increase in multidrug-resistant microorganisms (MDROs) on a global scale. Carbapenems are among the last antimicrobials used for treating infections caused by Enterobacterales, *Pseudomonas aeruginosa*, and *Acinetobacter baumannii* complex (ABC). The broad spectrum of β-lactam hydrolysis by carbapenemases poses a significant threat to therapeutic options, including carbapenems [1]. Latin American countries reported a sustained increase in resistance in gram-negative bacteria from 2010 to 2019. Also, the Latin American Network for Antimicrobial Resistance Surveillance recently issued warnings about the emergence of previously uncommon carbapenemase-producing Enterobacterales in Latin America and the increase in the number of isolates expressing ≥1 carbapenemase [2, 3].

The coronavirus disease 2019 (COVID-19) pandemic posed an additional threat and placed pressure on the increase in AMR worldwide. The rapid increase in the number of COVID-19 cases overwhelmed health systems, creating a multifactorial problem. Hospitalization, especially in intensive care units (ICUs), increases the chances of healthcare-associated infections (HAIs). A significant increase in HAIs from 2019 to 2020 was reported, mainly associated with the rise in the use of invasive devices, such as mechanical ventilation and vascular catheters. Increased length of stay, human resource challenges, and other operational changes that limited the implementation and effectiveness of standard infection prevention practices also contributed to the increase in HAIs [2–4].

The use of antimicrobials for COVID-19 patients to treat potential bacterial pathogens has become a widely implemented empirical practice [5, 6]. In one study, antimicrobial prescribing was reported in 72% of patients admitted to hospitals and 94% of COVID-19 patients admitted to ICUs, despite the low incidence of superinfections (8% according to the Infectious Disease Society of America [7]) and secondary bacterial infections (10%–15%) [6–8]. In this context, improved AMR monitoring and data analyses are critical to evidence regional differences to allow public health policies and more efficient epidemiological measures against resistance and therapeutic planning [9]. In this study, our aim was to evaluate detection rates (DRs), temporal trends, and COVID-19 impact on the most common carbapenemase resistance genes in Enterobacterales, *P. aeruginosa*, and ABC recorded in public health databases in Brazil from 2015 to 2022.

## METHODS

### Bacterial Isolates

The State Public Health Laboratories Network–LACEN (SISLAB) receives clinical samples related to patient care, mainly carbapenem-resistant organisms (CROs), from state hospitals. The hospital size varies from small to quaternary. Surveillance isolates (eg, rectal swabs) were excluded from this database, and only 1 isolate per patient per year was included in the study. A CRO isolate is confirmed as a carbapenemase producer using molecular methods established at each state laboratory. If polymerase chain reaction (PCR) testing is not implemented locally, isolates are sent to a regional reference laboratory (RRL) or a national reference laboratory (NRL). The state public health laboratory in Paraná, Brazil (LACEN-PR), is an RRL and receives CRO isolates from hospitals in Paraná and 4 other states. LAPIH, located in Rio de Janeiro, Brazil, is an NRL that receives samples from state health laboratories from 18 additional states. All results are sent to the public laboratory information system for inclusion in the datasets.

### Database

We used a unified database composed of 3 public laboratory information system datasets (see Supplementary Material). The 3 datasets were merged into the final database after an anonymization procedure with unique numeric identifications. The database was verified for duplicate samples. The main variables included in the database were date/year, microorganism (genus/species), resistance genes tested, and results. The database was composed of isolates received from January 2015 to September 2022.

### Carbapenemase Molecular Detection

All CROs received by RRLs and NRLs were submitted for molecular analysis using conventional or quantitative PCR testing to detect multiple carbapenemases for the following genes: Enterobacterales: *bla*_KPC_, *bla*_NDM_, *bla*_OXA-48_, *bla*_IMP_, *bla*_VIM_; *P. aeruginosa*: *bla*_SPM_, *bla*_KPC_, *bla*_NDM_, *bla*_IMP_, and *bla*_VIM_; and ABC: *bla*_KPC_, *bla*_NDM_, *bla*_OXA-23_, *bla*_IMP_, *bla*_VIM_, *bla*_OXA-24_, *bla*_OXA-58_, and *bla*_OXA-143_.

Different PCR protocols are used at each state’s public health laboratory, RRL, and NRL and have been validated independently.

### Statistical Analyses

The DR was obtained by dividing the number of genes detected by gene tested per microorganism and per year. The DR time trend was analyzed using the Prais–Winsten model [10], which creates a line of best fit between the time series points by linear regression and establishes the quantitative trend of a rate. The trend considers the serial correlation of the model errors using the logarithm of the observed rate values to reduce the heterogeneity of the variance of the regression analysis residuals. The time trend of the rate is presented as an annual percentage change (APC) with 95% confidence intervals (95% CIs). The APC is classified as stationary, increasing, or decreasing. The impact of the COVID-19 pandemic was evaluated by comparing the frequency of each antimicrobial resistance gene (ARG) detection pre- and post-pandemic onset, which was defined as 1 October 2017 to 31 March 2020 and 1 April 2020 to 22 September 2022, respectively. For the latter analysis, only LACEN-PR and NRL databases contained within this timeframe were used (see Supplementary Material). Frequency comparison was performed using the *χ*^2^ test, and *P* < .05 was considered significant. All analyses were performed with Stata 17.0 (StataCorp, College Station, TX).

## RESULTS

During the study period, 83 282 *bla*_KPC_ were tested in all 3 gram-negative groups. The Enterobacterales DR was 68.6% (41 282 of 60 205; Table 1). Considering all groups, 86 038 *bla*_NDM_ were tested, with an Enterobacterales DR of 14.4% (8391 of 58 172), 0.4% (63 of 15 338) for ABC, and 2.5% (309 of 12 528) for *P. aeruginosa*. The highest DR for ARGs was *bla*_OXA-23_ (92.2%, 15 218 of 16 505) in ABC, and the lowest DR was *bla*_VIM_ in ABC at 0.07% (2 of 2841).

**Table 1.** Distribution of Resistance Genes Detected/Tested Over the Study Period

Microorganism/microorganism group	Carbaopenemase gene detected	Study Year								
		2015	2016	2017	2018	2019	2020	2021	2022	Total
Enterobacterales	*bla*_KPC_	74.5% 6042/ 8116	76.3% 5278/ 6916	72.3% 4978/6882	71.0% 7068/ 9948	65.6% 6754/10,290	61.6% 7128/ 11 574	64.6% 3148/4871	55.1% 886/1608	68.6% 41 282/60 205
	*bla*_NDM_	4.1% 312/7700	4.7% 322/6816	8.2% 542/6596	8.7% 830/9500	15.7% 1532/9750	25.9% 2930/11 330	26.5% 1289/4872	39.4% 634/1608	14.4% 8391/58 172
	*bla*_IMP_	0% 0/0	40.0% 4/10	25.0% 6/24	0% 0/60	1.4% 4/288	1.4% 18/1272	5.7% 2/35	0% 0/16	2.0% 34/1705
	*bla*_VIM_	0% 0/0	0% 0/2	25% 4/16	12.5% 6/48	0.7% 2/278	0.2% 2/1280	8.5% 4/47	11.1% 2/18	1.2% 20/1689
	*bla*_OXA-48_	3.1% 124/3986	0.7% 16/2210	0.5% 8/1606	0.7% 32/4346	0.1% 4/4204	0.4% 18/5036	0.7% 8/1121	2.8% 4/141	0.9% 214/22 650
*Pseudomonas aeruginosa*	*bla*_KPC_	2.5% 40/1576	5.0% 50/1008	9.6% 102/1064	7.1% 98/1386	6.7% 142/2136	10.0% 320/3188	14.1% 219/1554	13.2% 94/713	8.4% 1065/12 625
	*bla*_NDM_	0.3% 4/1570	0% 0/1010	0% 0/1062	0.4% 6/1370	1.2% 24/2078	2.8% 90/3182	8.8% 136/1546	6.9% 49/710	2.5% 309/12 528
	*bla*_SPM_	22.5% 402/1786	11.8% 146/1232	9.0% 122/1350	11.2% 164/1460	9.3% 194/2074	4.1% 126/3084	4.5% 70/1546	4.0% 28/708	9.5% 1252/13 240
	*bla*_IMP_	0% 0/0	7.3% 16/218	0% 0/106	6.0% 14/234	12.0% 140/1212	11.0% 160/1450	4.5% 44/971	5.1% 33/648	8.4% 407/4839
	*bla*_VIM_	0% 0/0	0% 0/74	3.8% 22/582	7.8% 68/870	6.3% 130/2052	11.9% 370/3098	8.7% 133/1523	13.4% 95/708	9.2% 818/8907
*Acinetobacter baumannii*	*bla*_KPC_	0.7% 14/2082	0.5% 8/1572	0.2% 2/930	0.5% 6/1312	0.5% 8/1554	0.4% 8/2106	0.5% 4/831	3.1% 2/65	0.5% 52/10,452
	*bla*_NDM_	0.8% 16/2078	0.4% 6/1570	0.2% 2/1232	0% 0/1804	0% 0/2304	0.3% 8/2814	1.0% 28/2804	0.4% 3/732	0.4% 63/15 338
	*bla*_IMP_	0% 0/0	0% 0/0	0% 0/2	0% 0/10	33.3% 2/6	3.3% 2/60	0% 0/5	0% 0/4	4.6% 4/87
	*bla*_VIM_	0% 0/0	0% 0/0	0% 0/0	0% 0/2	0% 0/2	0.3% 2/622	0% 0/1948	0% 0/267	0.1% 2/2841
	*bla*_OXA-23_	96.5% 1950/2020	91.9% 1620/1762	86.5% 1254/1450	89.5% 1790/2000	89.5% 2302/2572	91.6% 2916/3182	97.1% 2710/2790	92.7% 676/729	92.2% 15 218/16 505
	*bla*_OXA-24_	0% 0/0	100% 2/2	8.9% 36/406	6.4% 50/784	4.5% 32/706	6.8% 30/440	47.4% 9/19	33.3% 1/3	6.8% 160/2360
	*bla*_OXA-58_	0% 0/0	0% 0/0	0% 0/406	0.5% 4/798	5.4% 40/742	16.4% 104/636	38.9% 7/18	0% 0/3	6.0% 155/2603
	*bla*_OXA-143_	30% 24/80	5.1% 28/548	13.1% 70/536	10.7% 94/ 878	5.5% 82/1486	6.9% 154/2246	2.7% 22/820	8.6% 5/58	7.2% 479/6652

### Detection Rate Temporal Trend

**Figure 1.**
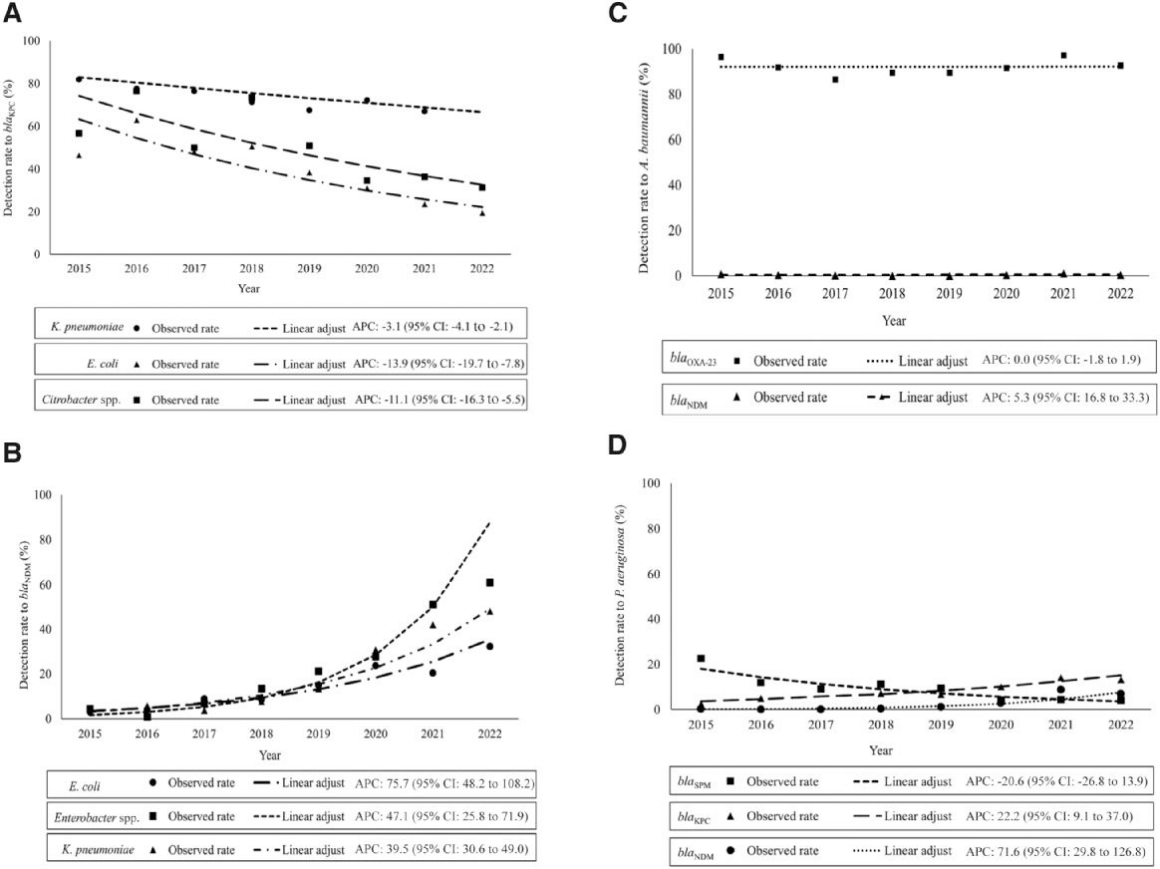
Temporal trend pattern for the detection rate of *bla*_NDM_ and *bla*_KPC_ in Enterobacterales, *Acinetobacter baumannii* complex, and *Pseudomonas aeruginosa*. Temporal trend pattern (APC) for *Klebsiella pneumoniae*, *Escherichia coli*, and *Citrobacter* spp. resistance to gene *bla*_KPC_*(A)*; *E. coli*, *Enterobacter* spp., and *K. pneumoniae* resistance to gene *bla*_NDM_*(B)*; *A. baumannii* resistance to *bla*_OXA-23_ and *bla*_NDM_*(C)*; and *P. aeruginosa* resistance to *bla*_SPM_, *bla*_KPC_, and *bla*_NDM_*(D)*. Abbreviations: APC, annual percentage change; CI, confidence interval.

During the period considered for the temporal trend, the Enterobacterales DR of *bla*_KPC_ decreased from 74.5% in 2015 to 55.1% in 2022 (4.0% APC decline; 95% CI, −4.8% to −3.3%). Furthermore, the Enterobacterales DR of *bla*_NDM_ increased from 4.1% in 2015 to 39.4% in 2022 (41.1% APC increase; 95% CI, 35.8% to 46.6%), all driven by the Enterobacterales species tested. These increases became more evident starting in 2017 (Tables 1 and 2, Figure 1*B* ), with a peak in 2022 for *Escherichia coli*, *Enterobacter* spp., and *Klebsiella pneumoniae* with annual increases of 75.7%, 47.1%, and 39.5%, respectively (Table 2, Figure 1*B* ). The Enterobacterales DRs for *bla*_VIM_, *bla*_IMP_, and *bla*_OXA-48_ were stationary. However, specific Enterobacterales resistance trends could not be tested for all ARGs. In summary, the most relevant findings relate to the decreasing trend in the DR of *bla*_KPC_ for Enterobacterales and the increasing trend in the DR for *bla*_NDM_ for Enterobacterales over time.

**Table 2.** Number of Genes Tested, Detection Rate, and Annual Percent Change From 2015 to 2022 for Each Gene Detection Rate Among the Main Enterobacterales, *Acinetobacter baumannii* Complex, and *Pseudomonas aeruginosa*

Microorganism	Gene	Tested (n)	Detected (%)	Annual Percent Change	95% Confidence Interval	Pattern
Enterobacterales	*bla*_KPC_	60 205	68.6	−4.0	**−4.8 to −3.3**	Decreasing
*Klebsiella pneumoniae*		41 224	74.7	−3.1	**−4.1 to −2.1**	Decreasing
*Escherichia coli*		2466	42.2	−13.9	**−19.7 to −7.8**	Decreasing
*Citrobacter* spp.		647	49.0	−11.1	**−16.3 to −5.5**	Decreasing
*Enterobacter* spp.		4573	49.2	−14.6	−27.5 to .6	Stationary
*Serratia* spp.		3240	64.4	−3.3	−9.9 to 3.7	Stationary
Enterobacterales	*bla*_NDM_	58 172	14.4	41.1	**35.8 to 46.6**	Increasing
*K. pneumoniae*		40 407	13.1	39.5	**30.6 to 49**	Increasing
*E. coli*		2390	18.4	75.7	**48.2 to 108.2**	Increasing
*Citrobacter* spp.		635	43.1	27.7	**22.2 to 33.4**	Increasing
*Enterobacter spp.*		4479	15.1	47.1	**25.8 to 71.9**	Increasing
*Serratia* spp.		3203	5.9	75.1	**28.4 to 138.7**	Increasing
Enterobacterales	*bla*_IMP_	1705	2.0	−42.4	−77.6 to 47.8	Stationary
Enterobacterales	*bla*_VIM_	1689	1.2	−16.9	−83.2 to 310.3	Stationary
Enterobacterales	*bla*_OXA-48_	22 650	0.9	−3.8	−42.7 to 61.6	Stationary
*K. pneumoniae*		14 536	1.3	−9.6	−42.5 to 42.2	Stationary
*Acinetobacter baumannii*	*bla*_KPC_	10 452	0.5	15.1	−11.3 to 49.4	Stationary
	*bla*_NDM_	15 338	0.4	5.3	−16.8 to 33.3	Stationary
	*bla*_OXA-23_	16 505	92.2	0.0	−1.8 to 1.9	Stationary
	*bla*_OXA-24_	2360	6.8	−4.3	−100 to 97.5	Stationary
	*bla*_OXA-58_	2603	5.9	302.9	**71 to 849.5**	Increasing
	*bla*_OXA-143_	6652	7.2	−15.7	**−23.8 to −6.8**	Decreasing
*Pseudomonas aeruginosa*	*bla*_KPC_	12 625	8.4	22.2	**9.1 to 37**	Increasing
	*bla*_NDM_	12 528	2.5	71.6	**29.8 to 126.8**	Increasing
	*bla*_IMP_	4839	8.4	−5.8	−25.7 to 19.7	Stationary
	*bla*_VIM_	8907	9.2	20.0	**11.8 to 28.7**	Increasing
	*bla*_SPM_	1 324	9.5	−20.6	**−26.8 to −13.9**	Decreasing

Bold values are for increasing or decreasing numbers.

ABC resistance remained stationary over time for most genes. The ABC DR temporal trend for *bla*_IMP_ and *bla*_VIM_ could not be tested. For *P. aeruginosa*, *bla*_SPM_ decreased from 22.5% in 2015 to 3.9% in 2022 (20.6% APC decline; 95% CI, −26.8% to −13.9%) and was the only gene with resistance reduction over time (Table 2). The *P. aeruginosa* DR temporal trend for *bla*_NDM_ had the highest APC observed in this species (71.6% increase; 95% CI, 29.8% to 126.8%). Figure 1 shows the most representative ARGs for the temporal trend of microorganisms. For *P. aeruginosa*, it is relevant to note the rise in *bla*_NDM_ and the decrease in *bla*_SPM_ over time.

### Impact of the COVID-19 Pandemic on Bacterial Resistance Genes

Compared with pre-pandemic onset, the Enterobacterales DR of *bla*_KPC_ increased from 57.1% to 61.8% in the post-onset period. Within the same time frame, *bla*_NDM_ in Enterobacterales increased from 18.7% to 28.0% (*P* < .001) and increased in all main species studied, *E. coli*, *Enterobacter* spp., and *K. pneumoniae*, with the DR reaching 51.3%, 43.0%, and 21.9% (*P* < .001). The ABC showed an increase from 0.4% to 0.7% in *bla*_NDM_ and from 91.9% to 95.8% in *bla*_OXA-23_. The *P. aeruginosa* DR increased from 8.8% to 11.8% for *bla*_KPC_ and from 1.1% to 6.8% for *bla*_NDM_ during the post-pandemic onset period. *bla*_SPM_ and *bla*_IMP_ for *P. aeruginosa* were the only genes with a reduced DR (Table 3). It is important to note that during the post-pandemic onset period, there was an increase in the detection of *bla*_KPC_ and *bla*_NDM_ in Enterobacterales, although the temporal trend of *bla*_KPC_ decreased over the 7-year period.

**Table 3.** Detection Rate for Resistance Genes During the Pre-Onset and Post-Onset Periods of the Coronavirus Disease 2019 Pandemic

	October 2017 to March 2020	April 2020 to September 2022	
Microorganism/Gene	% (n Tested / n Detected)	% (n Tested / n Detected)	*P* Value
Increased detection on rate			
Enterobacterales *bla*_KPC_	57.1% (3539/6201)	61.8% (5872/9506)	**<.001**
*Klebsiella pneumoniae bla*_KPC_	66.7% (3097/4646)	70.9% (5246/7395)	**<.001**
*Citrobacter* spp*. bla*_KPC_	19.1% (20/105)	33.0% (62/188)	**.011**
Enterobacterales *bla*_NDM_	18.7% (1158/6203)	28.0% (2664/9507)	**<.001**
*K. pneumoniae bla*_NDM_	14.4% (668/4646)	21.9% (1621/7393)	**<.001**
*Escherichia coli bla*_NDM_	36.0% (151/420)	51.3% (192/374)	**<.001**
*Enterobacter* spp*. bla*_NDM_	23.5% (158/672)	43.0% (299/696)	**<.001**
*Serratia* spp*. bla*_NDM_	8.4% (13/155)	29.7% (89/300)	**<.001**
*Acinetobacter baumannii bla*_OXA-23_	91.9% (2726/2965)	95.8% (4770/4979)	**<.001**
*Pseudomonas aeruginosa bla*_KPC_	8.8% (176/2002)	11.8% (386/3265)	**<.001**
*P. aeruginosa bla*_VIM_	7.8% (121/1546)	10.2% (331/3228)	**.007**
*P. aeruginosa bla*_NDM_	1.1% (22/1997)	6.8% (222/3255)	**<.001**
Decreased detection on rate			
*P. aeruginosa bla*_SPM_	7.5% (150/1998)	4.1% (134/3251)	**<.001**
*P. aeruginosa bla*_IMP_	9.0% (83/926)	6.0% (116/2077)	**.001**
Did not change			
*E. coli bla*_KPC_	21.0% (88/420)	19.5% (73/374)	.616
*Enterobacter* spp*. bla*_KPC_	28.0% (188/671)	24.9% (173/694)	.196
*Serratia* spp*. bla*_KPC_	64.9% (100/154)	68.0% (204/300)	.511
*Citrobacter* spp*. bla*_NDM_	65.7% (69/105)	69.2% (130/188)	.546
*A. baumannii bla*_KPC_	0.1% (1/862)	0.5% (7/1456)	.148
*A. baumannii bla*_NDM_	0.4% (11/2896)	0.7% (35/4999)	.072
*A. baumannii bla*_OXA-24_	58.3% (14/24)	48.5% (16/33)	.062
*A. baumannii bla*_OXA-58_	0% (0/14)	22.6% (7/31)	.053

Bold values are for increasing or decreasing numbers.

## DISCUSSION

Since the start of the 21st century, gram-negative bacteria have become an increasing problem as they relate to MDRO in Brazil, especially carbapenemase-producing organisms [11]. This situation became critical during the COVID-19 pandemic [2, 3], which has been the most severe pandemic of this century, causing 6 588 769 deaths worldwide; of those, 687 962 (10.4%) were in Brazil [12]. The death rate in Brazil was 4 times higher than the global median (319.5 × 82.5 deaths/100 k habitants globally) [13, 14].

In cooperation with the National Health Surveillance Agency (ANVISA—Portuguese acronym) and the Pan America Health Organization, the Brazilian Ministry of Health has made efforts to detect and control AMR since 2005 by establishing the AMR Network [15]. In 2015 for AMR Net, ANVISA chose 4 RRLs in 4 Brazilian states (Paraná, São Paulo, Brasília, Piauí) and 1 NRL (LAPIH- Fundação Oswaldo Cruz Rio de Janeiro) to provide AMR referral testing in healthcare services [16]. In 2018, Brazil started participating in the World Health Organization–Global Antimicrobial Resistance Surveillance System (GLASS) and published its National Acting Plan on AMR, which established the need to create a national surveillance program on AMR (BR-GLASS) [15, 17–19]. By 2020, the BR-GLASS database contained more than 30 000 isolates. After an international call from the US Centers for Disease Control and Prevention as part of the Global Antimicrobial Resistance Laboratory and Response Network, the General Coordination of Public Health Laboratories (Minister of Health (MoH)) proposed a new program, the Strengthening of the Brazilian Surveillance System on AMR, supported by multiple national and state partners.

Because of these initiatives and better structuring of the Brazilian AMR Net, the analysis included here represents results from the molecular detection of carbapenemases at SISLAB from 2015 to 2022 in Brazil. Here, we demonstrate the substantial increase in the prevalence of carbapenemase genes during the post-pandemic onset period (2020–22) compared with the pre-onset period (2017–2020).

Although we observed an increase in carbapenemase production, the resistance rate of carbapenem from primary bloodstream infections that were reported to ANVISA through the national program for the prevention and control of HAIs was very similar when we compared pre- and post-pandemic onset periods, even though this report analyzed data up to 2021 [20].

Since it was first described in 2006 [21], *bla*_KPC_ has become one of the most worrisome resistance genes among Enterobacterales in Brazil. Many outbreaks have been described in Brazil. Since 2015, it has reached an endemic state, as shown in our study, as well as in many countries of Latin America, as described by PAHO at the CARBA-LA Project (Pillonetto et al, 2023, manuscript in preparation). Since 2015, our data have shown that the *bla*_KPC_ DR seems to be decreasing for Enterobacterales; it has also decreased for *K. pneumoniae*, *Citrobacter* spp., and *E. coli*. The same decrease in *bla*_KPC_ was found in a Brazilian study published by Wink et al [22]. Several factors may explain the declines observed in *bla*_KPC_. First, some Brazilian hospitals are using methods for the detection of *bla*_KPC_ (phenotypically or genotypically) and no longer refer *bla*_KPC_-positive isolates to the reference laboratories for confirmation because they consider *bla*_KPC_ to be endemic. Second, a larger number of isolates were tested for *bla*_KPC_, including strains that were polymyxin-resistant but not necessarily carbapenem-resistant. Finally, the increase in *bla*_NDM_ detection, as demonstrated in the present analysis, could mean a possible replacement of carbapenemases in Brazil. This situation was also noted by Arend et al who showed that the increase in NDM-producing bacteria in southern Brazil was probably due to the presence of this gene in different plasmids [23]. Comparing the pre- and post-pandemic onset periods and considering the database for LACEN-PR and NRL only, an increase in the KPC DR of 5% was observed within the Enterobacterales order. The same was observed for the main species that produce *bla*_KPC_, *K. pneumoniae.* However, our analysis shows that not only was the *bla*_KPC_ DR rising during the post-pandemic onset period but also the total amount of strains sent to the reference laboratories for Enterobacterales and for *K. pneumoniae*, where the total isolates tested increased by more than 3000 and the positive tests surpassed more than 2300 isolates (see Table 3).

In addition to the high endemicity for *bla*_KPC_ in Brazil, *bla*_NDM_ was first detected in Enterobacterales, in 2012 in *Enterobacter hormaechei* and in 2013 in *Providencia rettgeri* [24, 25]. Our study showed that the Enterobacterales DR for *bla*_NDM_ increased consistently from 2015 to 2022 (from 4.2% to 23.8%), becoming more evident starting in 2017, with its peak in the pandemic years (2020–2021, mainly in 2022). Also, the total amount of *bla*_NDM_ detected in Enterobacterales rose in more than 1500 isolates comparing pre- and post- pandemic onset (see Table 3).

Accordingly, da Silva et al [26] reported 81 *bla*_NDM_ cases in 9 states, 4 in 2012–2013, 27 in 2014, and 50 in 2015. Also, Thomas et al [3] observed an increase in *bla*_NDM_ in many Latin American countries, which is supported by the PAHO CARBA-LA project, which includes data from 12 countries from 2015 to 2020 (Pillonetto et al, 2023, personal communication). The rise in *bla*_NDM_ cannot be explained by a selective pressure caused by the use of the newer β-lactam/ β-lactamase inhibitor because this class of drugs has a very high cost for low- and middle-income countries such as Brazil and other countries in Latin America. Consequently, its use is very restricted. Also, most of the recently published studies, including ours, show a more evident increase from 2018 on, peaking during the pandemic years (2020–2022) [3, 22]. One hypothesis for the higher increase in the pandemic years compared with the pre-pandemic years is the clonal expansion related to overcrowded hospitals, hiring unprepared health professionals, and the indiscriminate use of antibiotics, as shown in some studies with up to 94% of COVID-19–infected patients receiving antimicrobials, especially broad-spectrum drugs [27].

The detection of the main carbapenemase gene in ABC (*bla*_OXA-23_) started in Brazil during the first outbreak that was reported globally in 1999 [28]. However, a continuous increase over the last 2 decades was seen in Brazil, where the clonality and carbapenem resistance kept spreading [29, 30]. Although we did not observe a significant change in *bla*_OXA-23_ detection over our study period, a 4% increase of the *bla*_OXA-23_ DR was seen during the post-pandemic onset period, with an additional 2000 *bla*_OXA-23_ isolates of ABC strains detected at the reference laboratories. The increase in carbapenem-resistant *A. baumannii* (CRAB) that we observed during the post-pandemic onset period has been reported in other studies [27]. At least 2 outbreaks of CRAB were reported in Brazil during the pandemic. Shinohara et al [31] reported 14 isolates in 1 ICU, and Camargo et al [32] found 224 patients colonized or infected by international clone 2, which is relatively uncommon in Brazil. Polly et al [33] showed a significant increase (+108.1%) in incidence density (ID) of MDR infections by CRAB in all hospitals when comparing pre-pandemic and pandemic periods and a 48% increase in ICUs ID for CRAB during the pandemic.

Although much less common than *bla*_OXA-23_, the presence of *bla*_NDM_ in *Acinetobacter* spp. is another interesting finding, with the first Brazilian isolate detected in *Acinetobacter pittii* in September 2012 [34] followed by the detection in 2014 in *A. baumannii* [35], *Acinetobacter bereziniae* [36], and *Acinetobacter nosocomialis* [37].

*Pseudomonas aeruginosa*’s primary mechanisms of resistance to carbapenems are overexpression of the efflux pump and overproduction of AmpC β-lactamase, which is associated with the inactivation of the OprD outer membrane protein. However, the production of carbapenemases has played an increasing role in this species [38]. Although many carbapenemase genes have been described globally in *P. aeruginosa*, the *bla*_VIM_ and *bla*_IMP_ genes are the most prevalent [38]. From 2015 to 2020 in 12 Latin American countries, *bla*_VIM_ and *bla*_KPC_ were detected at 52.2% and 22.4%, respectively (Pillonetto M et al, 2023, personal communication). Until recently, *bla*_SPM_ was the most prevalent ARG in Brazilian *P. aeruginosa* isolates. This gene was first isolated in 1997 in São Paulo, Brazil [39], and has since been detected in almost every region of Brazil [40, 41]. The peak of *bla*_SPM_ detection in Brazil occurred between 2000 and 2012 and was associated with a single clone, ST277. However, in the last 10 years, a decrease in the prevalence of *bla*_SPM_ and a higher frequency of isolation of other carbapenemases, mainly *bla*_KPC_, *bla*_NDM_, and *bla*_VIM_, have been observed in some hospitals in Brazil [42]. Our present study corroborates these findings (APCs of *bla*_SPM_: −20.6, *bla*_VIM_: 19.9, *bla*_KPC_: 22.2, and *bla*_NDM_: 71.6). One possible explanation for this change in the profile of carbapenemase production in *P. aeruginosa* is the presence in Brazil of high-risk multidrug-resistant clones, such as ST233 and ST244, which carry carbapenemases such as *bla*_KPC_ and *bla*_VIM_ [43, 44]. An important rise in the total *P. aeruginosa* strains sent to the reference laboratories (from 12 404 to 19 013, 65.3%) and a significant increase in *bla*_NDM_ during the post-pandemic onset period were observed, with the DR rising from 1% in the pre-onset period to 7% in the post-onset period. Perez et al [45] also found NDM-producing *P. aeruginosa* in 27 of 156 (17.3%) patients during the COVID-19 pandemic.

To our knowledge, this is the first study in Brazil to compile data from 27 state and federal districts. A main strength of our study is the total number of strains studied (more than 80 000) over a long period of time (>7 years), including pre- and post-pandemic onset. No duplicates or surveillance swabs were included. The study did have limitations. There was no unique protocol for all states regarding referral of samples to the state reference laboratories. Also, there is no guarantee that all state reference laboratories used the same PCR protocol for ARG detection and that the protocols for receiving and investigating ARGs did not change during the study period. Some reference laboratories experienced an overload of isolates during the post-pandemic period, and stricter rules had to be implemented to limit the number of samples received. This action could have caused an underestimation of the overall increase in ARGs during the COVID-19 pandemic. Finally, the total number of hospitals that sent isolates to the reference laboratories could not be accessed.

This study shows the strengths of the Brazilian AMR Surveillance Network, with robust data related to carbapenemases in Brazil over time and the impact of COVID-19. The data clearly show an increase in total isolates sent to the reference laboratories, especially from 2020 to 2022. Additionally, we observed a tendency for the modification of carbapenemases profiles, mainly with an important annual rise in *bla*_NDM_ over the study period. It is unclear if ARGs will continue to increase steadily after the pandemic. However, this could have a direct impact on the use of new carbapenemase inhibitor drugs since they have no effect on metallo- β-lactamase. All of the AMR surveillance data from Brazil will be of great importance in enhancing projects that are already underway to improve the Brazilian health system and its AMR efforts and coordinate new and stronger actions for identifying risk factors for the spread of MDROs with adequate allocation of resources and policies intended to improve the diagnosis, prevention, and treatment in the country.

## Supplementary Data

Supplementary materials are available at *Clinical Infectious Diseases* online. Consisting of data provided by the authors to benefit the reader, the posted materials are not copyedited and are the sole responsibility of the authors, so questions or comments should be addressed to the corresponding author.

## Supplementary Material

ciad260_Supplementary_Data

## Notes

***Acknowledgments.*** The authors thank Gisele Bernardi Aparecida, Geiziane Gonçalves, and Thais Gaio for technical support and Ricardo Bergamo for information technology and database support.

***Financial support.*** The Strengthening of the Brazilian Surveillance System on AMR [Antimicrobial Resistance] program is funded by the US Centers for Diseases Control and Prevention (CDC; CDC-RFA-CK21-2104).

***Supplement sponsorship***. This article appears as part of the supplement “The Evolving Challenges of Antibiotic Resistance in Low- and Middle-Income Countries: Priorities and Solutions,” sponsored by the U.S. Centers for Disease Control and Prevention, and Health Security Partners.
