# Supplementary material for: A 7-Year Brazilian National Perspective on Plasmid-Mediated Carbapenem Resistance in Enterobacterales, Pseudomonas aeruginosa, and Acinetobacter baumannii Complex and the Impact of the Coronavirus Disease 2019 Pandemic on Their Occurrence
Source: Clin Infect Dis. 2023 Jul 5;77(Suppl 1):S29–37. doi: 10.1093/cid/ciad260 (PMC10321697; doi:10.1093/cid/ciad260)
Supplement: ciad260_Supplementary_Data [file ciad260_supplementary_data.docx]

Supplementary material

**A seven-year Brazilian national perspective on plasmid-mediated carbapenem resistance in Enterobacterales, *Pseudomonas aeruginosa*, and *Acinetobacter baumannii* and the impact of the Covid-19 pandemic on their occurrence.**

Carlos R. V. Kiffer, Thais F. T. Rezende, Daniela Testoni Costa-Nobre, Ana Sílvia Scavacini Marinonio, Lucas Hidemitsu Shiguenaga, Debora Nicole Oliveira Kulek, Lavinia Nery Villa Stangler Arend, Ivson Cassiano de Oliveira Santos, Bruna Ribeiro Sued-Karam, Claudio Marcos Rocha-de-Souza, Leticia Kraft, Andre Abreu, Renata Tigulini de Souza Peral, Ana Paula D’Alincourt Carvalho-Assef*, Marcelo Pillonetto*

* These co-authors are both senior authors and contributed equally to this manuscript.

**Supplementary Methods**

The present study used three sets of original data provided by public laboratory information systems: National GAL (“Gerenciador de Ambiente Laboratorial”- Laboratory Environment Manager), LACEN-PR (Laboratório Central do Paraná) and NRL (National Reference Laboratory).

GAL is a dataset developed for public health laboratories by the Ministry of Health to collect SISLAB (State Public Health Laboratories Network) data for epidemiological surveillance and includes variables with information regarding patient identification and demographic characteristics, characteristics of isolate collection (date, sample clinic, microorganism, antibiotic resistance with methods and results for each) tested resistance genes and results. The database contained one row per tested microorganism and was available for the study from January 1, 2015 to December 31, 2020.

LACEN-PR and NRL are part of SISLAB, and both generate data that feed the National GAL system. The LACEN-PR and NRL datasets contained information on patient identification and characteristics, collection location (laboratory, unit, municipality), isolated collection characteristics (collection date, id, clinical specimen, laboratory dates, microorganism) and methods and results for tested resistance genes. Both databases contained one row per sample with more than one microorganism per row. LACEN-PR and NRL were converted to one microorganism per line. All information was available from January 1, 2017 to September 22, 2022.

The three received datasets were merged into a final database. An anonymization procedure was followed with unique numeric IDs. After these procedures, the final unified database included: year, microorganism, tested resistance genes and resistance gene results.

The detection rates (DR) and the temporal trend analysis (Prais Winsten model) were generated from data of the final unified database, which contained GAL data from 2015-2020 and LACEN-PR/NRL data from 2021-2022. Only LACEN-PR and NRL data from 2017 to 2022 were used to assess the impact of COVID-19 (chi-square test), since GAL data did not contain 2021 and 2022.
